# Incidence of Appendicitis over Time: A Comparative Analysis of an Administrative Healthcare Database and a Pathology-Proven Appendicitis Registry

**DOI:** 10.1371/journal.pone.0165161

**Published:** 2016-11-07

**Authors:** Stephanie Coward, Hashim Kareemi, Fiona Clement, Scott Zimmer, Elijah Dixon, Chad G. Ball, Steven J. Heitman, Mark Swain, Subrata Ghosh, Gilaad G. Kaplan

**Affiliations:** 1 Department of Medicine, University of Calgary, Calgary, AB, Canada; 2 Community Health Sciences, University of Calgary, Calgary, AB, Canada; 3 Information Management and Dissemination, Medicine, Alberta Health Services, Calgary, Alberta, Canada; 4 Department of Surgery, University of Calgary, Calgary, AB, Canada; University of Illinois-Chicago, UNITED STATES

## Abstract

**Importance:**

At the turn of the 21^st^ century, studies evaluating the change in incidence of appendicitis over time have reported inconsistent findings.

**Objectives:**

We compared the differences in the incidence of appendicitis derived from a pathology registry versus an administrative database in order to validate coding in administrative databases and establish temporal trends in the incidence of appendicitis.

**Design:**

We conducted a population-based comparative cohort study to identify all individuals with appendicitis from 2000 to2008.

**Setting & Participants:**

Two population-based data sources were used to identify cases of appendicitis: 1) a pathology registry (n = 8,822); and 2) a hospital discharge abstract database (n = 10,453).

**Intervention & Main Outcome:**

The administrative database was compared to the pathology registry for the following *a priori* analyses: 1) to calculate the positive predictive value (PPV) of administrative codes; 2) to compare the annual incidence of appendicitis; and 3) to assess differences in temporal trends. Temporal trends were assessed using a generalized linear model that assumed a Poisson distribution and reported as an annual percent change (APC) with 95% confidence intervals (CI). Analyses were stratified by perforated and non-perforated appendicitis.

**Results:**

The administrative database (PPV = 83.0%) overestimated the incidence of appendicitis (100.3 per 100,000) when compared to the pathology registry (84.2 per 100,000). Codes for perforated appendicitis were not reliable (PPV = 52.4%) leading to overestimation in the incidence of perforated appendicitis in the administrative database (34.8 per 100,000) as compared to the pathology registry (19.4 per 100,000). The incidence of appendicitis significantly increased over time in both the administrative database (APC = 2.1%; 95% CI: 1.3, 2.8) and pathology registry (APC = 4.1; 95% CI: 3.1, 5.0).

**Conclusion & Relevance:**

The administrative database overestimated the incidence of appendicitis, particularly among perforated appendicitis. Therefore, studies utilizing administrative data to analyze perforated appendicitis should be interpreted cautiously.

## Introduction

The appendectomy for appendicitis is the most commonly performed emergency abdominal operation conducted in North America. Approximately one-third of patients with appendicitis will experience a perforation of their appendix before their appendectomy.[1–3] Perforated appendicitis is more likely to lead to sepsis, in-hospital complications, and mortality when compared to non-perforated appendicitis. Studies that have separately studied the incidence of perforated and non-perforated appendicitis have inconsistently reported that the incidence of non-perforated and perforated appendicitis is changing over time.[4–6]

However, many epidemiologic studies of appendicitis have relied on administrative healthcare databases to study appendicitis and to differentiate perforated from non-perforated appendicitis.[6–10] These studies have used discharge abstract databases that code appendicitis based on the *International Classification of Disease*, *Ninth Revision*, *Clinical Modification* (ICD-9-CM) or the *Tenth Revision* (ICD-10-CA).[11, 12] Reliance on ICD codes to identify cases with appendicitis introduces the potential for a misclassification error because approximately 12% of appendectomies remove a normal appendix.[13] This error can potentially be compounded when codes are used to differentiate appendicitis into perforated versus non-perforated appendicitis. Misclassification of ICD codes may result in misreporting of the incidence of appendicitis and possibly incorrect conclusions when assessing temporal trends of incidence.[14] These issues are negated when a pathology-proven registry of appendicitis cases are used; however, pathology reports are not available for research in most jurisdictions and compiling this data is time- and cost-inefficient.

Thus, the objective of this manuscript was to compare appendicitis cases, stratified by perforated and non-perforated, derived from an administrative healthcare database to a cohort derived from a pathology-confirmed registry in order to validate ICD coding of appendicitis and to evaluate the effect of misclassification on temporal trend analyses of incidence.

## Materials and Methods

A population-based study was conducted in the Calgary Health Zone (CHZ) to identify all individuals (adults and children) who were admitted to hospital for appendicitis between January 1, 2000 and December 31, 2008.

### Data Sources

We used two data sources to identify patients with appendicitis: 1) a pathology proven cohort of patients with appendicitis derived from the Calgary Lab Services (CLS) database; and 2) a hospital discharge abstract database derived from the Data Integration Management and Reporting (DIMR) administrative healthcare database. The pathology proven cohort was used to define the study population, whereas the administrative database was used to supplement clinical information on the cohort.

The CLS database was used to attain the pathology reports of all appendix specimens resected from hospitals within the CHZ from 2000 to 2008. The CLS database contains free-text searchable fields. The original histopathology slides were not available for review and thus, the pathology reports were manually reviewed to confirm the diagnosis of appendicitis and to stratify the cases into non-perforated or perforated appendicitis based on the interpretation provided by general surgeons (ED, CB). Data extraction of the pathology reports were performed independently by two investigators (SC, HK).

The DIMR hospital discharge abstract database was used to identify all individuals in the CHZ who were admitted to hospital for acute appendicitis from 2000 to 2008. These patients were coded for non-perforated appendicitis (ICD-9-CM: 540.9; or ICD-10-CA: K35.1, K35.9) or perforated appendicitis (ICD-9-CM: 540.0, 540.1; or ICD-10-CA: K35.0).[11, 12] The administrative healthcare database was screened for duplicate admissions (e.g., interval appendectomy); the date of appendicitis for patients with multiple admissions was based on date of appendectomy derived from the pathology report. An individual patient with appendicitis was only counted once in our analyses.

The patients from DIMR and CLS databases were matched based on their unique personal health number, date of birth, and date of surgery. Matching was done to supplement the pathology report that was attained from the CLS database with DIMR administrative hospital data that included age, sex, residence, and comorbidities. Patients admitted to hospital for appendicitis, but living outside of the CHZ were excluded. The process of linking the DIMR and CLS databases has been previously described.[15]

### Study Population

**Fig 1:**
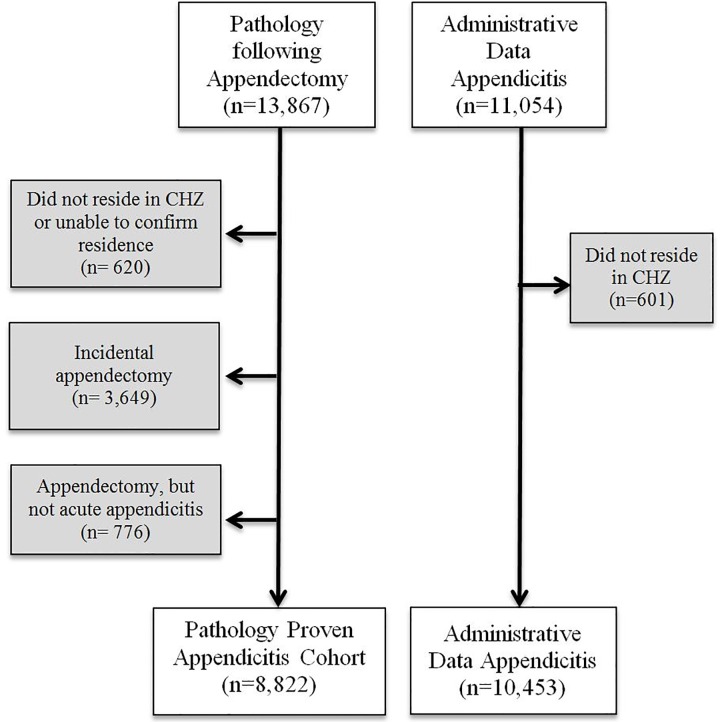
Flow-chart of the study populations derived for the pathology proven registry and the administrative healthcare database.

We searched for all pathology reports with the term “appendectomy” or “appendicitis”, which identified 13,867 pathology reports from the CLS database. Pathology reports were manually reviewed to exclude patients with an incidental appendectomy or a diagnosis other than appendicitis leaving 9,442 cases pathology-proven acute appendicitis. These cases were matched to the DIMR database to determine residence at time of hospital admission. Appendicitis cases living outside of the CHZ, or residence could not be confirmed, were excluded (n = 620). The final population included 8,822 cases of pathology-proven appendicitis (Fig 1).

A separate cohort was derived from the DIMR administrative healthcare database to identify patients with an ICD code for appendicitis (n = 11,054). After excluding patients living outside of the CHZ (n = 601), the final study population was 10,453 patients (Fig 1).

### Statistical Analysis

The cohort of patients with appendicitis derived from the pathology proven reports was used to define the study population. We calculated the annual incidence of appendicitis for the following scenarios: 1) all appendicitis cases; 2) adult (≥18 years old) versus pediatric (<18 years); and 3) perforated versus non-perforated appendicitis. Incidence was calculated by dividing the number of new cases of appendicitis by the population size of the CHZ, which assumed a Poisson distribution to calculate 95% confidence intervals (CI). Temporal trend analyses for the annual incidence of appendicitis from 2000 to 2008 were assessed using a generalized linear model that assumed a Poisson distribution, and the CI was calculated using robust variance adjustment. The annual percent change (APC) with 95% CI was calculated for all cases of appendicitis, adult appendicitis versus pediatric, and non-perforated versus perforated appendicitis. Age and sex standardized incidence and APCs were calculated for pathology-proven appendicitis using the 2006 Canada Census population as the reference.

Next we calculated the incidence of appendicitis using the data derived solely from the DIMR administrative healthcare database using ICD codes to identify the study population. Incidence was calculated for all cases, age groups, and perforation status. We compared the incidence of appendicitis derived from an administrative database to those derived from the pathology registry. This comparative analysis was conducted to demonstrate the differences in reported incidence data derived from administrative databases without confirmation by pathology reports. To explain the differences between incidences in the administrative versus pathology cohorts we calculated the positive predictive value (PPV) with 95% CI for the ICD codes used to identify appendicitis in the DIMR database. The PPV was evaluated annually from 2000 to 2008, and the APC for the change in PPV over time was evaluated using a linear regression model.

## Results

**Fig 2:**
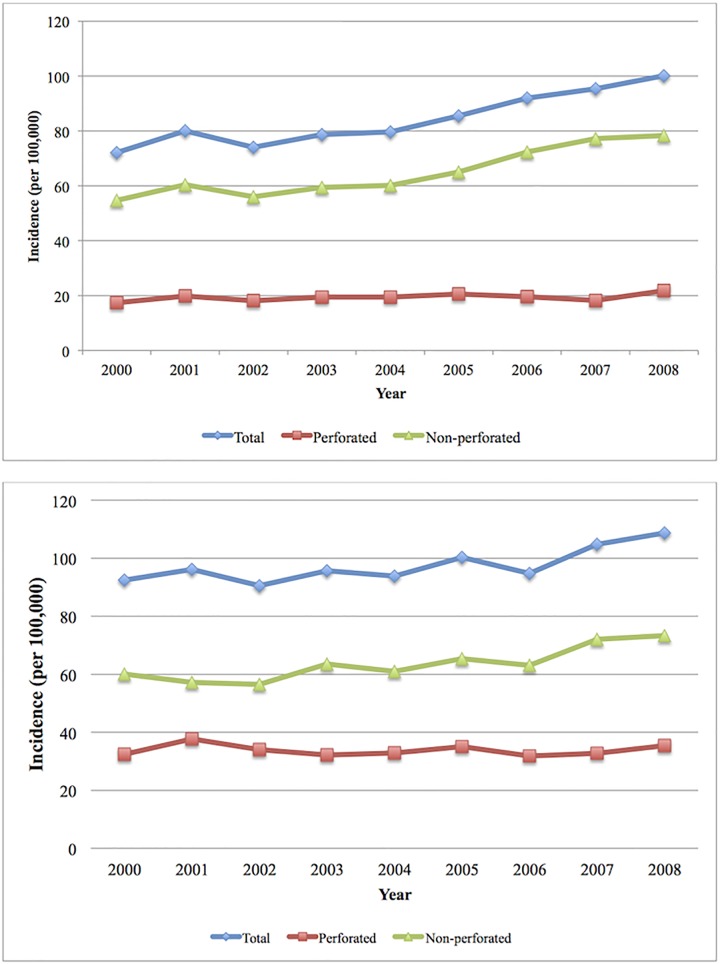
Annual incidence of appendicitis stratified by perforated and non-perforated from cohorts derived by A) a pathology proven registry; and B) an administrative healthcare database.

Table 1 provides the characteristics of patients with appendicitis derived from the pathology-proven registry and the administrative database. The average annual incidence of appendicitis based on the pathology-proven registry was 84.2, 64.9, and 19.4 per 100,000 persons for all cases, non-perforated appendicitis, and perforated appendicitis, respectively (Table 2). Incidence of appendicitis was higher in males as compared to females (S1 Table). The annual incidence of appendicitis, stratified by perforated and non-perforated status, is displayed in Fig 2 for both the pathology-proven registry (Fig 2A) and the administrative healthcare database (Fig 2B). The administrative healthcare database overestimated the incidence of appendicitis when compared to the pathology proven database for all appendicitis cases, pediatric-onset, adult-onset, and perforated appendicitis (Table 2). Age and sex standardized incidence for pathology-proven appendicitis is reported in S2 Table.

**Table 1:** Clinical characteristics of patients with appendicitis derived from a pathology-proven registry and from an administrative healthcare database.

Characteristics	Pathology Proven Registry (n = 8,822)	Administrative Database (n = 10,453)	P-value
**Age, % (n)**			
0–17	22.9 (2,021)	26.1 (2,724)	<0.001
18–31	30.6 (2,700)	29.8 (3,117)	0.23
32–47	26.1 (2,303)	24.7 (2,586)	0.03
48+	20.4 (1,798)	19.4 (2,026)	0.08
**Sex, % (n)**			
Male	54.1 (4,771)	54.0 (5,645)	0.89
Female	45.9 (4,051)	46.0 (4,808)	0.89
**Comorbidity, % (n)**			
0 Comorbidities	94.5 (8,332)	94.3 (9,860)	0.55
1 Comorbidities	4.1 (366)	4.2 (437)	0.73
>2 Comorbidities	1.4 (124)	1.5 (156)	0.56
**Perforation Cohort, % (n)**			
Non-perforated	77.1 (6.803)	65.4 (6,833)	<0.001
Perforated	22.9 (2,019)	34.6 (3,620)	<0.001

**Table 2:** Comparative analysis in incidence and temporal trends between cohorts of appendicitis patients derived from a pathology-proven registry and an administrative healthcare database.

	Positive Predictive Value (95% CI)	Pathology Proven Registry		Administrative Database	
		Annual Incidence (n)	APC (95% CI)	Annual Incidence (n)	APC (95% CI)
**All Appendicitis**	83.0% (82.2, 83.7)	84.2 per 100,000 (8,822)	4.1 (3.1, 5.0)	100.3 per 100,000 (10,453)	2.1(1.3, 2.8)
**Perforated Appendicitis**	52.4% (50.6, 54.2)	19.4 per 100,000 (2,019)	1.4 (-0.3, 3.2)	34.8 per 100,000 (3,620)	0.1 (-1.2, 1.4)
**Non-perforated Appendicitis**	92.7% (92.0, 93.4)	64.9 per 100,000 (6,803)	4.9 (3.8, 6.0)	65.3 per 100,000 (6,833)	3.3 (2.3, 4.2)
**Pediatric Appendicitis**	73.0% (71.3–74.7)	82.8 per 100,000 (2,021)	3.0 (1.5, 4.4)	112.6 per 100,000 (2,724)	0.3 (-1.2, 1.7)
**Adult Appendicitis**	86.5% (85.7, 87.2)	84.6 per 100,000 (6,801)	4.4 (3.3, 5.6)	96.5 per 100,000 (7,729)	2.8 (1.9, 3.7)

APC–annual percent change; CI–confidence interval.

From 2000 to 2008 the incidence of appendicitis was significantly increasing every year for cohorts derived from the pathology-proven registry (APC = 4.1%; 95% CI: 3.1, 5.0) and administrative database (APC = 2.1%; 95% CI: 1.3, 2.8). Using the pathology-proven registry the incidence of non-perforated appendicitis increased by 4.9% per year (95% CI: 3.8, 6.0), but was stable for perforated appendicitis (APC = 1.4%; 95% CI: -0.3, 3.2). The pathology proven database showed that the incidence of appendicitis was increasing in children (APC = 3.0%; 95% CI: 1.5, 4.4); in contrast, the administrative database incorrectly found that the incidence of appendicitis was stable for pediatric patients (APC = 0.3%; 95% CI: -1.2, 1.7) (Table 2). The APC were similar for males as compared to females (S1 Table).

The PPV for ICD coding of appendicitis in the administrative health databases was 83.0% (95% CI: 82.2% to 83.7%) for all appendicitis cases. The PPV was lower for ICD codes identifying perforated (PPV = 52.4%; 95% CI: 50.6%-54.2%) as compared to non-perforated appendicitis (PPV = 92.7%; 95% CI: 92.0%-93.4%) (Table 2). When comparing the validity of perforated appendicitis codes in identifying any case of true appendicitis (perforated or non-perforated) the PPV increases to 84.1% (95% CI: 82.9%, 85.3%). The PPVs were similar for males as compared to females (S1 Table). Overtime, the PPV increased from 74.6% in 2000 to 88.7% in 2008 (APC = 1.44; 95% CI: 1.04, 1.85) (Table 3).

**Table 3:** Positive predictive value of coding in the administrative healthcare database as compared to the pathology-proven registry stratified overtime.

Year	Positive Predictive Value (95% Confidence Interval)
2000	74.6% (71.8, 77.3)
2001	81.6% (79.1, 83.9)
2002	79.8% (77.2, 82.3)
2003	80.6% (78.1, 72.9)
2004	81.7% (79.2, 83.9)
2005	83.3% (81.1, 85.4)
2006	85.0% (83.0, 87.0)
2007	87.3% (85.4, 89.1)
2008	88.7% (86.9, 90.3)

## Discussion

Since appendicitis entered the medical vernacular in 1885, the incidence of appendicitis in North America and Europe has risen dramatically.[16] In the middle of the 20^th^ century, the incidence of appendicitis was consistently reported to be decreasing.[17, 18] However, studies reporting temporal trends in the incidence of appendicitis during the latter part of the 20^th^ century have reported inconsistent findings.[18, 19] Particularly, studies that separately evaluated incidence rates stratified by perforated and non-perforated appendicitis have shown divergent patterns.[6] One potential explanation for the heterogeneity of findings in more recent papers has been the greater reliance of administrative healthcare databases to capture appendicitis cases, as opposed to patient registries where diagnosis has been confirmed. Our study confirms that ICD coding of appendicitis misclassifies a subset of appendicitis cases and that these misclassification errors can influence the annual incidence rates and temporal trend analyses.

A systematic review on the incidence of appendicitis demonstrated that since 1990 the majority of studies in North America and Europe used administrative databases to measure incidence.[18] For example, since 2000 only one population-based cohort published in North America confirmed the diagnosis of appendicitis.[20] The incidence of appendicitis, since 1990, in North America and Europe ranged from 75 to 150 per 100,000 persons. In contrast, population-based studies in North America and Europe that evaluated medical registries with confirmation of diagnosis reported that since 1990 the incidence of appendicitis ranged from 44 to 84 per 100,000.[18] Similarly, in our study the incidence of appendicitis was overestimated by the administrative healthcare database by nearly 15%. Our findings have clinical and public relevance because of the reliance on administrative healthcare databases for appendicitis research evaluating clinical outcomes, incidence, healthcare delivery, and costs.

The overestimation of the incidence of appendicitis is predominantly explained by misclassification errors as demonstrated by an overall PPV of 83%. Further, our study demonstrated that this misclassifying of a normal appendectomy was accentuated in children diagnosed with appendicitis. One explanation for the misclassification error was that some cases of normal or incidental appendectomy were falsely recorded as appendicitis.[13] In contrast, the incidence of appendicitis derived from the pathology-proven registry would not capture patients with appendicitis treated non-operatively with antibiotics or milder cases that spontaneously resolve. In our administrative database we identified 255 patients (2.4%) who were coded for appendicitis, but lacked a corresponding procedural code for an appendectomy and did not have a pathology report. These patients likely represented medically managed appendicitis.

The administrative healthcare database accurately identified non-perforated appendicitis (PPV = 93%), which led to reporting of similar incidences (~64 per 100,000) in both the pathology-proven registry and administrative database. In contrast, nearly half of the perforated appendicitis cases were misclassified leading to an incidence of perforated appendicitis that was nearly two times higher in the administrative database cohort. Visualizing the appendix during laparoscopic surgery can be difficult and may result in a misdiagnosis of perforated appendicitis.[21] However, in some cases the pathology is reported after the patient is discharged from hospital, which may lead to discordance between the discharge abstract database and the pathology report. Regardless of the reason for the discrepancy, this highlights that administrative databases reporting on incidence or outcomes of perforated appendicitis should be interpreted cautiously and ideally be locally validated. Future studies should be directed at developing coding algorithms for appendicitis that reduce misclassification errors in administrative databases.

Since 1990, temporal trend analyses on the incidence of appendicitis in North America have been inconsistently reported with studies demonstrating increased[6, 22, 23] and decreased[2, 24] incidences. In part, the divergence in temporal trend analyses of appendicitis studies may be explained by misclassifications errors that change across time. For example, the PPV in our study substantially improved across the course of study period with a PPV of 74.6% in 2000 that rose to 88.7% in 2008. If the proportion of false positives coded as appendicitis in an administrative healthcare database decreases overtime this would lead to an artificial decline in the incidence of appendicitis. This would explain why the APC of incidence for appendicitis was half the value for the administrative database (2% per year) as compared to the pathology-proven registry (4% per year). This artificial decline in incidence over time stemming from administrative database studies has been proposed in other conditions such as inflammatory bowel disease.[25]

Further, studies have also reported divergent incidences between perforated and non-perforated appendicitis. Overall, we observed rising incidence of appendicitis, which was mainly driven by the increasing incidence of non-perforated appendicitis. Our administrative database accurately predicted the temporal trends (i.e., APC) detected in the pathology-proven registry, including those for all appendicitis cases, and stratified by perforated and non-perforated. However, the administrative database failed to detect the rising incidence of appendicitis in children. Thus, methodological limitations explain some, but not all, of the differences observed in our study as compared to other population-based incidence studies.

For example, a study using administrative data from Ontario, Canada showed that the rate of non-perforated appendicitis was decreasing, whereas perforated appendicitis was increasing.[2] Thus, differences in temporal trends observed in other regions are likely explained by multiple factors. Diagnostic studies, such as enhanced use of CT scans, have altered the landscape for appendicitis and led to fewer normal appendectomies.[13, 26] Also, the routine performance of incidental appendectomies has fallen out of clinical practice.[27] While the evolution in the diagnosis and management of appendicitis has likely altered the incidence of appendicitis over time, these practices may be employed differently in separate regions of North America. Alternatively, differential exposures to environmental risk factors of appendicitis (e.g. fiber consumption) may result in variable incidence rates across different geographic areas[28]. Future studies are necessary to explain differences in temporal trends observed across the globe.

This study has several strengths including population-based design, large sample size, and manual review of pathology reports. However, several limitations should be considered. First, the administrative database that was validated may not be generalizable to other administrative databases used to capture appendicitis hospitalizations. However, the discharge abstract database is designed to support the Canadian Institute for Health Information and thus, is standardized for reporting of hospitalizations across Canada. Second, approximately 100 cases of appendicitis were identified in the pathology registry, but were not identified in the discharge abstract administrative database. Because we used the administrative database to determine city of residence at time of admission we were not able to determine whether these cases lived in the Calgary Health Zone and were excluded from the incidence analysis. Third, we calculated the PPV of the administrative database in order to explore the effect of false positive cases in administrative databases; however, we did not evaluate the sensitivity or specificity of the codes. Also, we did not assess codes associated with chronic or recurrent appendicitis (e.g., K36) and unclassified appendicitis (e.g., K37). Thus, some cases of acute appendicitis coded outside of K35 may have been missed. Further, we defined perforated and non-perforated appendicitis based on the interpretation of the pathology report without reviewing the histological slides.

Based on the pathology proven registry the incidence of appendicitis treated operatively was 84 per 100,000 and increased by over 4% per year. The rise in incidence was predominantly observed in non-perforated appendicitis and among pediatric-onset appendicitis. The explanation for the rising incidence may be explained by evolving diagnostic and management approaches for appendicitis. These data were subsequently compared to findings from an administrative database. This comparison is clinically relevant because most prior studies in North America used administrative databases to report the incidence of appendicitis. Based on our validation work, studies using administrative databases likely misclassified approximately 15% of cases with the greatest errors occurring in children and perforated appendicitis. Further, caution should be applied in interpreting temporal trend analyses among pediatric-onset appendicitis. Therefore, studies using administrative data to study appendicitis should be interpreted cautiously and, if possible, validated for accuracy of diagnosis. Finally, future studies should focus on developing coding algorithms that improve the accuracy of detecting appendicitis from administrative healthcare databases.

## Supporting Information

S1 Table Comparative analysis in incidence and temporal trends between cohorts of appendicitis patients derived from a pathology-proven registry and an administrative healthcare database stratified by male and female. (DOCX)

S2 Table Age and sex standardized incidence of appendicitis with annual percent change (APC). (DOCX)
