# Supplementary material for: Incidence of Appendicitis over Time: A Comparative Analysis of an Administrative Healthcare Database and a Pathology-Proven Appendicitis Registry
Source: PLoS One. 2016 Nov 7;11(11):e0165161. doi: 10.1371/journal.pone.0165161 (PMC5098829; doi:10.1371/journal.pone.0165161)
Supplement: S1 Table — (DOCX) [file pone.0165161.s001.docx]

**S1 Table: Comparative analysis in incidence and temporal trends between cohorts of appendicitis patients derived from a pathology-proven registry and an administrative healthcare database stratified by male and female.**

Male

|  | **Positive Predictive Value (95% CI)** | **Pathology-Proven Registry (CLS)** | | **Administrative Database (DIMR)** | |
| --- | --- | --- | --- | --- | --- |
|  |  | **Annual Incidence** | **APC (95% CI)** | **Annual Incidence** | **APC (95% CI)** |
| **All Appendicitis** | 83.3%  (82.4, 84.3) | 91.5 per 100,000 | 4.0 (2.8, 5.1) | 108.5 per 100,000 | 2.0 (1.0, 3.1) |
| **Perforated Appendicitis** | 52.6%  (50.2, 55.0) | 21.4 per 100,000 | 1.1 (-1.2, 3.4) | 38.7 per 100,000 | -0.1 (-1.8, 1.6) |
| **Non-perforated Appendicitis** | 92.7%  (91.7, 93.6) | 70.0 per 100,000 | 4.9 (3.6, 6.2) | 69.7 per 100,000 | 3.2 (1.9, 4.6) |
| **Pediatric**  **Appendicitis** | 73.7%  (71.4-75.8) | 93.0 per 100,000 | 3.2 (0.9, 5.6) | 125.1 per 100,000 | 0.4 (-1.5, 2.4) |
| **Adult Appendicitis** | 87.0%  (86.0, 88.1) | 90.9 per 100,000 | 4.3 (2.9, 5.6) | 103.2 per 100,000 | 2.7 (1.5, 4.0) |

Female

|  | **Positive Predictive Value (95% CI)** | **Pathology-Proven Registry (CLS)** | | **Administrative Database (DIMR)** | |
| --- | --- | --- | --- | --- | --- |
|  |  | **Annual Incidence** | **APC (95% CI)** | **Annual Incidence** | **APC (95% CI)** |
| **All Appendicitis** | 82.5%  (81.4, 83.6) | 77.1 per 100,000 | 4.2 (3.0, 5.5) | 91.8 per 100,000 | 2.3 (1.2, 3.4) |
| **Perforated Appendicitis** | 52.1%  (49.4, 54.8) | 17.3 per 100,000 | 1.8 (-0.7, 4.4) | 30.8 per 100,000 | 0.4 (-1.5, 2.3) |
| **Non-perforated Appendicitis** | 92.8%  (91.7, 93.8) | 59.8 per 100,000 | 4.9 (3.5, 6.4) | 60.9 per 100,000 | 3.3 (1.9, 4.7) |
| **Pediatric**  **Appendicitis** | 72.1%  (69.5, 74.7) | 72.0 per 100,000 | 2.7 (0.1, 5.4) | 97.9 per 100,000 | 0.7 (-1.5, 3.0) |
| **Adult Appendicitis** | 85.8%  (84.7, 87.0) | 78.6 per 100,000 | 4.6 (3.2, 6.1) | 89.9 per 100,000 | 2.8 (1.5, 4.1) |
