# Supplementary material for: Incidence of Appendicitis over Time: A Comparative Analysis of an Administrative Healthcare Database and a Pathology-Proven Appendicitis Registry
Source: PLoS One. 2016 Nov 7;11(11):e0165161. doi: 10.1371/journal.pone.0165161 (PMC5098829; doi:10.1371/journal.pone.0165161)
Supplement: S2 Table — (DOCX) [file pone.0165161.s002.docx]

**S2 Table: Age and sex standardized incidence of appendicitis with annual percent change (APC).**

|  | **Pathology-Proven Registry (CLS)** | | **Adjusted (Age and Sex)** | |
| --- | --- | --- | --- | --- |
|  | **Annual Incidence** | **APC (95% CI)** | **Annual Incidence** | **APC (95% CI)** |
| **All Appendicitis** | 84.2 per 100,000 | 4.1 (3.1, 5.0) | 84.3 per 100,000 | 3.89 (1.1, 6.8) |
| **Perforated Appendicitis** | 19.4 per 100,000 | 1.4 (-0.3, 3.2) | 19.4 per 100,000 | 1.33 (-4.3, 7.3) |
| **Non-perforated Appendicitis** | 64.9 per 100,000 | 4.9 (3.8, 6.0) | 64.4 per 100,000 | 5.1 (1.8, 8.5) |
| **Pediatric**  **Appendicitis** | 82.8 per 100,000 | 3.0 (1.5, 4.4) | 79.4 per 100,000 | 0.5 (-2.3, 3.4) |
| **Adult Appendicitis** | 84.6 per 100,000 | 4.4 (3.3, 5.6) | 84.1 per 100,000 | 4.6 (1.7, 7.5) |
